# Severe myocardium suppression in two congenital heart disease patients after remdesivir use – a case report

**DOI:** 10.1186/s12887-025-06315-y

**Published:** 2025-11-14

**Authors:** Yi-Fan Lin, Shih-Yu Fang, Shu-Chien Huang, En-Ting Wu

**Affiliations:** 1 Department of Pediatrics, National Taiwan University Children’s Hospital, Taipei, 100 Taiwan; 2 Department of Surgery, WanFang Hospital, Taipei, 116 Taiwan; 3 Department of Surgery, National Taiwan University Hospital, Taipei, 100 Taiwan; 4 Department of Pediatrics, National Taiwan University Children’s Hospital, No.8, Chung Shan S. Rd., Zhongzheng Dist, Taipei City, 10041 Taiwan

**Keywords:** Remdesivir, SARS-CoV-2, Congenital heart disease, Mitochondrial dysfunction, Cardiogenic shock

## Abstract

**Background:**

Remdesivir, the first FDA-approved antiviral for SARS-CoV-2, has shown clinical benefits in early COVID-19 but is associated with cardiac adverse events. Experimental studies indicate remdesivir can impair mitochondrial dynamics and alter cardiomyocyte electrophysiology. We report two pediatric patients with congenital heart disease (CHD) who developed profound cardiogenic shock shortly after remdesivir administration.

**Case presentations:**

Patient 1 was a 10-year-old boy with hypoplastic left heart syndrome admitted with COVID-19. Within hours of the first remdesivir infusion, he developed refractory hypotension, QRS widening, and cardiac arrest, ultimately requiring venoarterial extracorporeal membrane oxygenation (VA-ECMO). He later died from intracranial hemorrhage.

Patient 2 was a 15-year-old boy with repaired pulmonary atresia and 34 residual right ventricular failure. After two consecutive remdesivir doses, he 35 developed hypotension, conduction abnormalities, and refractory shock requiring 36 VA-ECMO. Despite temporary stabilization, he succumbed to multiorgan failure 37 and intra-abdominal infection.

**Discussion:**

Both patients were initially stable before infusion but developed shock within three hours, aligning with remdesivir pharmacokinetics. The temporal relationship, ECG changes, and elevated lactate suggest remdesivir-induced myocardial suppression, possibly mediated through mitochondrial fragmentation, impaired quality control, and UTS2R activation. These effects may have synergized with systemic inflammation, sepsis, and chronic ventricular overload in CHD, tipping myocardium already at the edge of decompensation.

**Conclusion:**

Although causality cannot be proven, these cases highlight a potential risk of remdesivir-induced myocardial suppression in children with complex CHD and systemic inflammation. We recommend careful risk–benefit assessment and cautious use of remdesivir in patients with single-ventricle physiology or right ventricular failure, especially when treatment is initiated late in the disease course.

## Background

Since the first outbreak in late 2019, SARS-CoV-2 has caused substantial morbidity and mortality worldwide [1]. Remdesivir (Veklury^®^), a nucleotide pro-drug whose active intracellular metabolite (GS-441524 triphosphate) inhibits the viral RNA-dependent RNA polymerase, was the first antiviral to receive full FDA approval for treating hospitalized COVID-19 patients [2]. Its indication has since been expanded to include pediatric patients, including neonates who meet specified weight- and age-based criteria.

Early randomized trials, such as the NIAID ACTT-1 study, demonstrated that remdesivir shortens time to recovery, particularly in patients requiring supplemental oxygen but not yet on invasive mechanical ventilation [2]. Network meta-analyses and Cochrane reviews suggest that remdesivir likely reduces the risk of clinical worsening (for example, progression to mechanical ventilation or death) in hospitalized patients with moderate to severe COVID-19 [3–6]. Retrospective studies indicate that early administration (within ~ 7 days of symptom onset) is associated with a reduced likelihood of ICU admission or need for mechanical ventilation especially in patients not yet requiring advanced respiratory support [7–9]. Guidelines from the American College of Physicians recommend remdesivir for hospitalized patients who have not yet required invasive ventilation or ECMO, noting modest improvements in recovery rate and decreased progression to more intensive respiratory support [10]. Pediatric pharmacokinetic and safety studies—including the CARAVAN program and Phase 2/3 trials—support its safe use across pediatric age groups (infants aged ≥ 28 days, weight-based doses). Exposure levels in children are comparable to those in adults [11–13]. 

From basic science, however, there is in vitro evidence that remdesivir can injure mitochondria in cardiomyocytes: mitochondrial fragmentation, reduced redox potential, suppressed mitochondrial respiration, sarcomere disarray, electrophysiological alterations, some lasting after drug cessation, and cell death. Mechanistic studies implicate excessive mitochondrial fission as a key contributor [14–16]. 

Clinically, remdesivir has been temporally associated with cardiovascular adverse events—most often sinus bradycardia—in both pediatric and adult COVID-19 patients [17, 18]. Case reports and small observational studies describe bradyarrhythmia, hypotension, QT prolongation, and other conduction abnormalities, often shortly after the loading dose of remdesivir [19, 20]. Retrospective analyses in children and adults have similarly reported increased odds of bradycardia or ECG changes temporally related to dosing [21, 22]. Nevertheless, large randomized controlled trials and meta-analyses have not demonstrated a statistically significant increase in serious cardiac adverse events (arrhythmias, myocardial injury, heart failure) in remdesivir-treated patients compared to standard care [3, 23, 24]. In pediatric studies, multiple retrospective studies and case series report no significant bradycardia, hypertension, or arrhythmias attributable to remdesivir, even among those with underlying cardiac disease. For instance, in a single-center study of 48 pediatric patients, no significant bradycardia or hypertension was observed [13]; in in a Japanese case series, 20% had cardiac disease, no serious cardiovascular adverse events were reported [25]. In Phase 2/3 trials, about 21% of participants had cardiac disorders, but no new safety signals or cardiovascular events emerged [11]. However, these studies often lack detailed description of the nature and severity of underlying cardiac disease and frequently do not specify whether the COVID-19 infection in these patients was moderate, severe, or critical. Importantly, there is currently no retrospective study in pediatric COVID-19 patients with CHD that directly compares the incidence of cardiovascular side effects between those treated with remdesivir and those who did not receive it.

Here, we report two pediatric patients with CHD who developed profound cardiogenic shock shortly after remdesivir administration. The temporal association suggests that remdesivir-induced mitochondrial dysfunction, potentially amplified under systemic inflammation, might contribute. We further propose that pathological mitochondrial adaptation in severe CHD renders patients especially vulnerable to remdesivir-induced myocardium suppression. While causality cannot be definitively established, these cases raise concern that remdesivir be used with caution in certain CHD population and underscore the need for further clinical investigation.

## Case presentation

Remdesivir was administered according to a standardized, weight-based dosing protocol in both patients. The drug was diluted in normal saline and delivered by intravenous infusion. For patients younger than 28 days, the regimen consisted of a 2.5 mg/kg loading dose on Day 1, followed by 1.25 mg/kg once daily. For patients weighing less than 40 kg and aged 28 days or older, the regimen consisted of a 5 mg/kg loading dose on Day 1, followed by 2.5 mg/kg once daily. For patients weighing more than 40 kg, the regimen included a 200 mg loading dose on Day 1, followed by 100 mg once daily. The infusion duration ranged from 30 to 120 min.

### Patient 1

This 10-year-old boy with hypoplastic left heart syndrome underwent the initial stage of the Norwood procedure shortly after birth, followed by the creation of a bidirectional Glenn shunt before reaching one year of age. Because of poor family support, he lost follow-up until 2021 when he presented with profound cyanosis with oxygen saturation 60%. He underwent palliative Blablock-Thomas-Taussig shunt (BT shunt) creation to rescue left pulmonary artery hypoplasia. Thereafter, the resting oxygen saturation elevated up to 75–80% and subnormal single ventricle ejection fraction (50%) was documented on echocardiography.

**Fig. 1:**
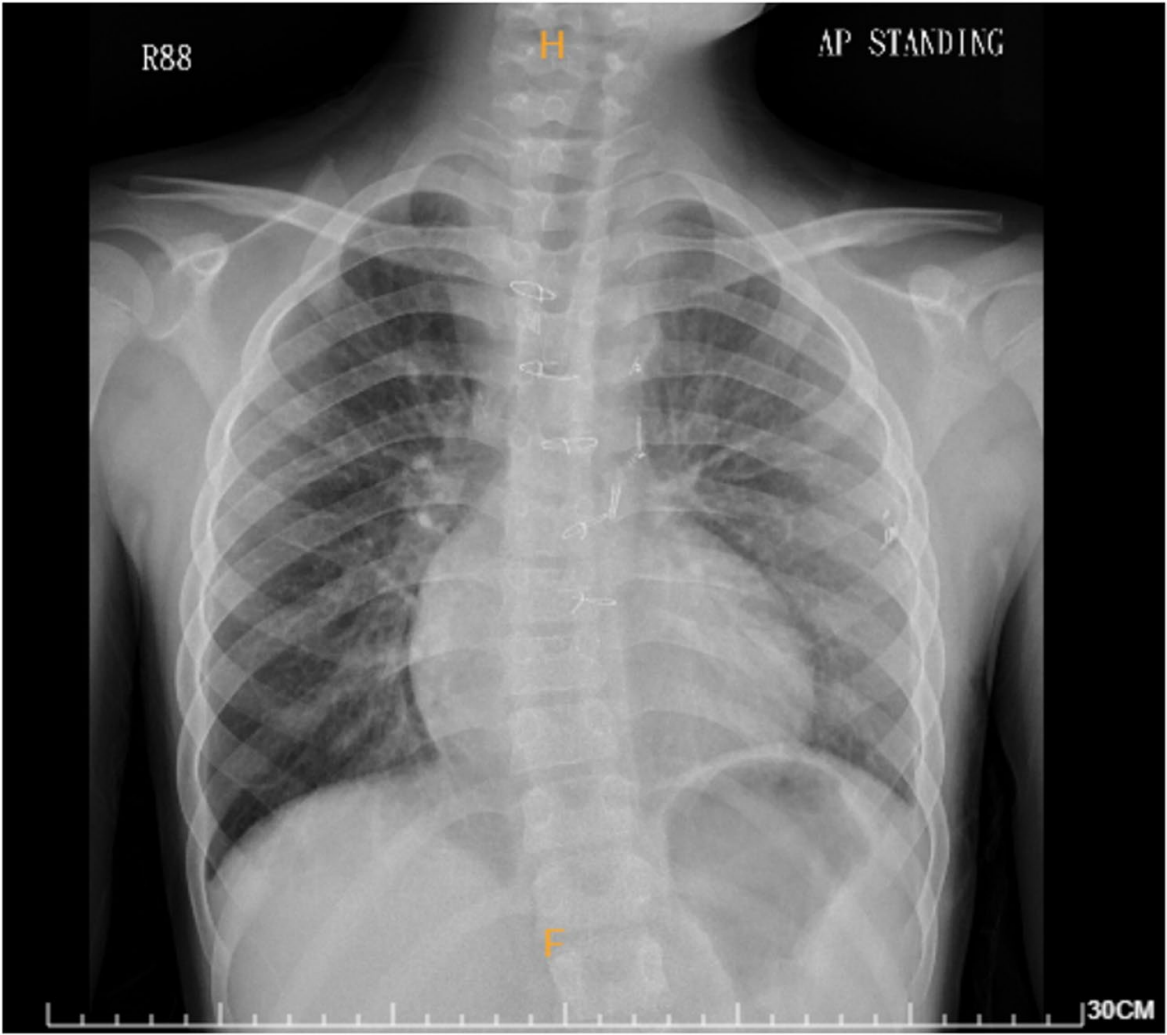
A chest radiograph taken 2 months before the current hospitalization showing enlargement of the cardiac silhouette on the right side

In late April 2022, the patient’s persistent productive cough escalated to dyspnea, leading to admission. SARS-CoV-2 was ruled out by the RT-PCR test, and chest radiography (CXR) showed no pneumonia but mild cardiomegaly (Fig. 1). Empirical treatment with Ampicillin/Sulbactam switched to Teicoplanin due to Methicillin-resistant Staphylococcus aureus in the blood culture. Subsequent blood cultures were negative, and the patient condition was stable. Breakthrough fever occurred on May 6th with blood culture yielded Citrobacter koseri, prompting Piperacillin/Tazobactam administration. Abdominal ultrasound revealed hydrops gallbladder, engorged hepatic vein, and portal vein, suggesting suboptimal right heart function. The abdominal computer tomography (CT) scan showed thick soft tissue infiltrates near the abdominal para-aortic region. Intra-abdominal infection (IAI) was highly suspected.

The patient encountered his family member infected with SARS-CoV-2. Progressive cough was noted since May 16th. The cycle threshold in RT-PCR test on May 19th was 18.53. A remdesivir infusion was initiated on the next day, administered over 90 min. Four hours later, oxygen saturation dropped to 20–30% despite escalation of respiratory support. ECG revealed a widening of the QRS duration. In addition, hypotension gradually developed and was refractory to vasopressor therapy. Subsequently, cardiac arrest occurred. He regained spontaneous circulation after 7 min of CPR. Bedside echocardiogram showed global hypokinesia. Central venous pressure (CVP) measured through inferior vena cava was up to 30 mmHg, and mixed venous oxygen saturation was 24%. VA-ECMO was then initiated for cardiogenic shock.

Under ECMO support, blood pressure improved, but lactatemia continued to progress. A peritoneal dialysis tube was inserted to relieve abdominal distension. Three days later, lactatemia declined and the echocardiogram showed recovered contractility. Two days later, the patient suffered from intracranial hemorrhage, with both pupils dilated and unresponsive to light. ECMO was withdrawn and the patient passed away.

**Fig. 2:**
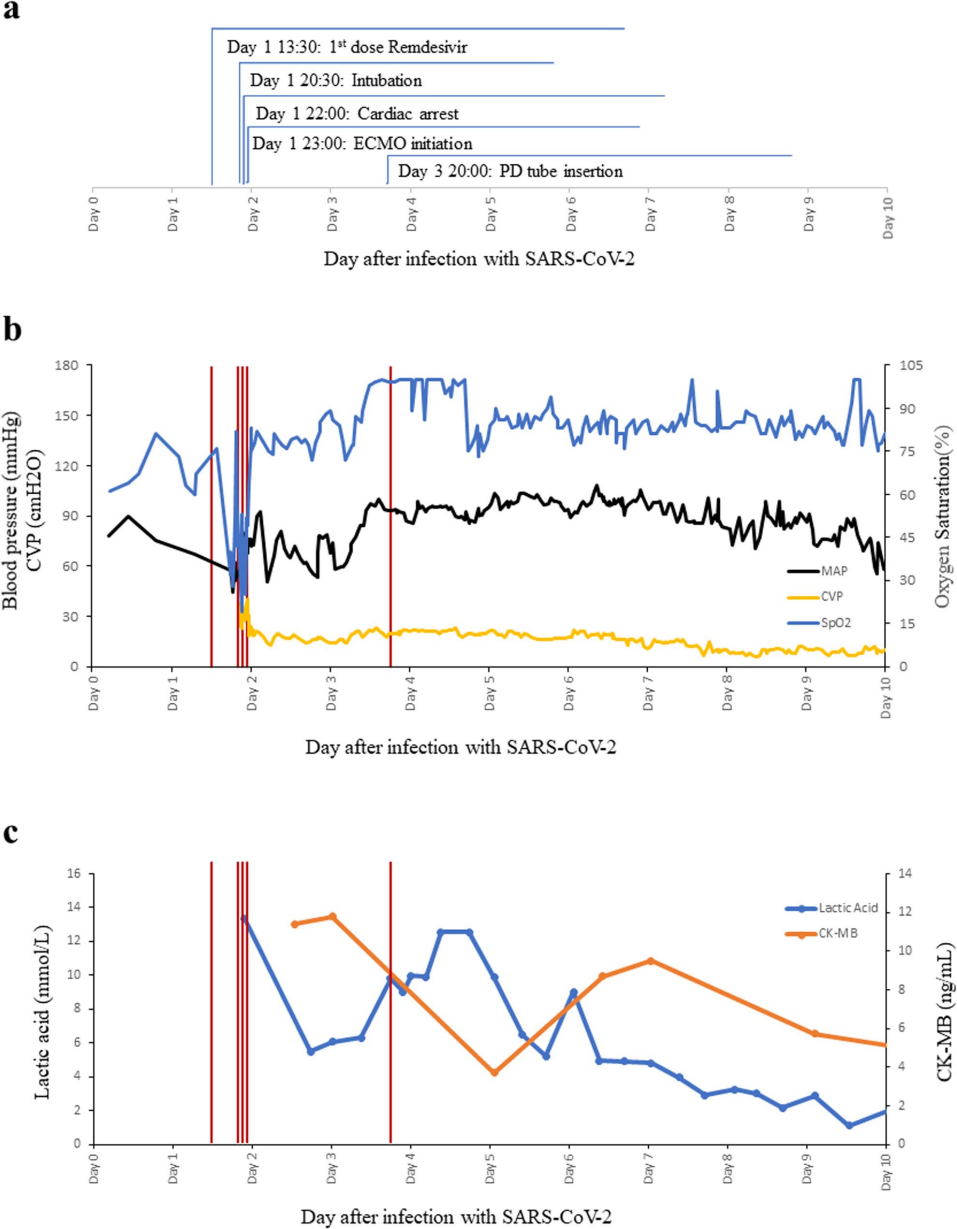
**a**-**c** Clinical details and changes in lab data over time in Patient 1 after infection with SARS-CoV-2 and the administration of remdesivir. Day 0 represents the day of confirmed SARS-CoV-2 infection. MAP, mean arterial pressure; CVP, central venous pressure; PD, peritoneal dialysis

The clinical details and changes in lab data over time in Patient 1 after infection with SARS-CoV-2 and the administration of remdesivir were summarized in Fig. 2a-c.

### Patient 2

**Fig. 3:**
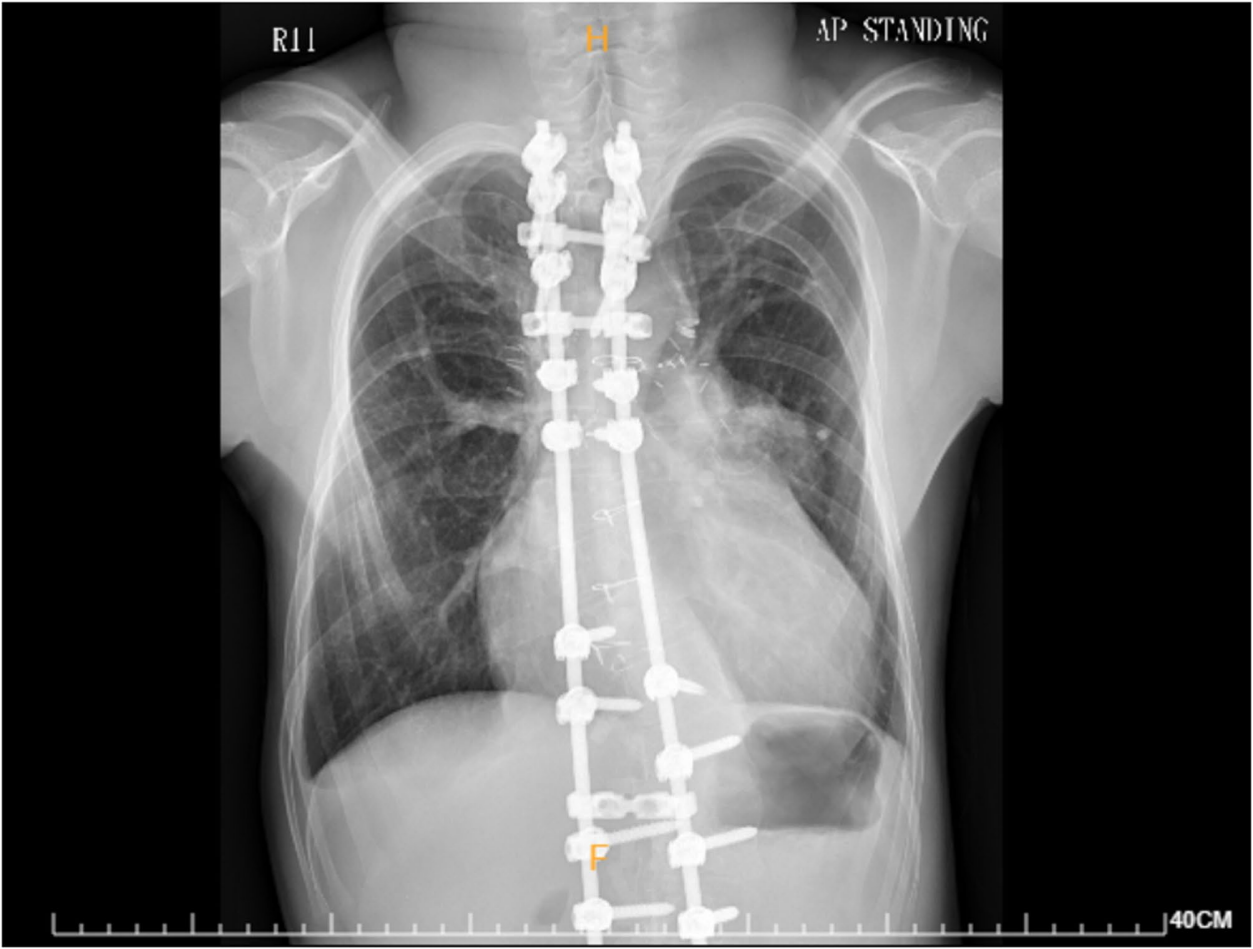
A chest radiograph taken 1 month before the current hospitalization, showing cardiomegaly and spinal rods implanted for correction of scoliosis

A 15-year-old boy was born with DiGeorge syndrome, pulmonary atresia, ventricular septal defect, and major aortopulmonary collateral arteries (MAPCAs). He underwent BT shunt creation, unifocalization of the MAPCAs, and complete septation with two-ventricle repair at the age of 11 years. Postoperatively, progressive right ventricle (RV) dilatation with significant residual pulmonary stenosis with pressure gradient up to 36 mmHg, indicating RV failure, was documented on echocardiography. CXR revealed cardiomegaly and patchy infiltrations. (Fig. 3) He also had a history of megacolon complicated with necrotizing enterocolitis, for which he had undergone subtotal colectomy with end-to-end ileocolonic anastomosis in his infancy, and early onset scoliosis, for which he had undergone posterior instrumentation with growing rod system.

The patient was admitted to the hospital because of SARS-CoV-2 infection and presented with a high fever, sore throat, cough, and loose stools for 3 days since September 28th, 2022. The cycle threshold in the SARS-CoV-2 RT-PCR test was 14.3, and his IL-6 levels had risen to 33,999, indicating high viral load and severe systemic inflammation. Empirical Piperacillin/Tazobactam was given for elevated levels of CRP and procalcitonin, which indicated bacterial co-infection.

**Fig. 4:**
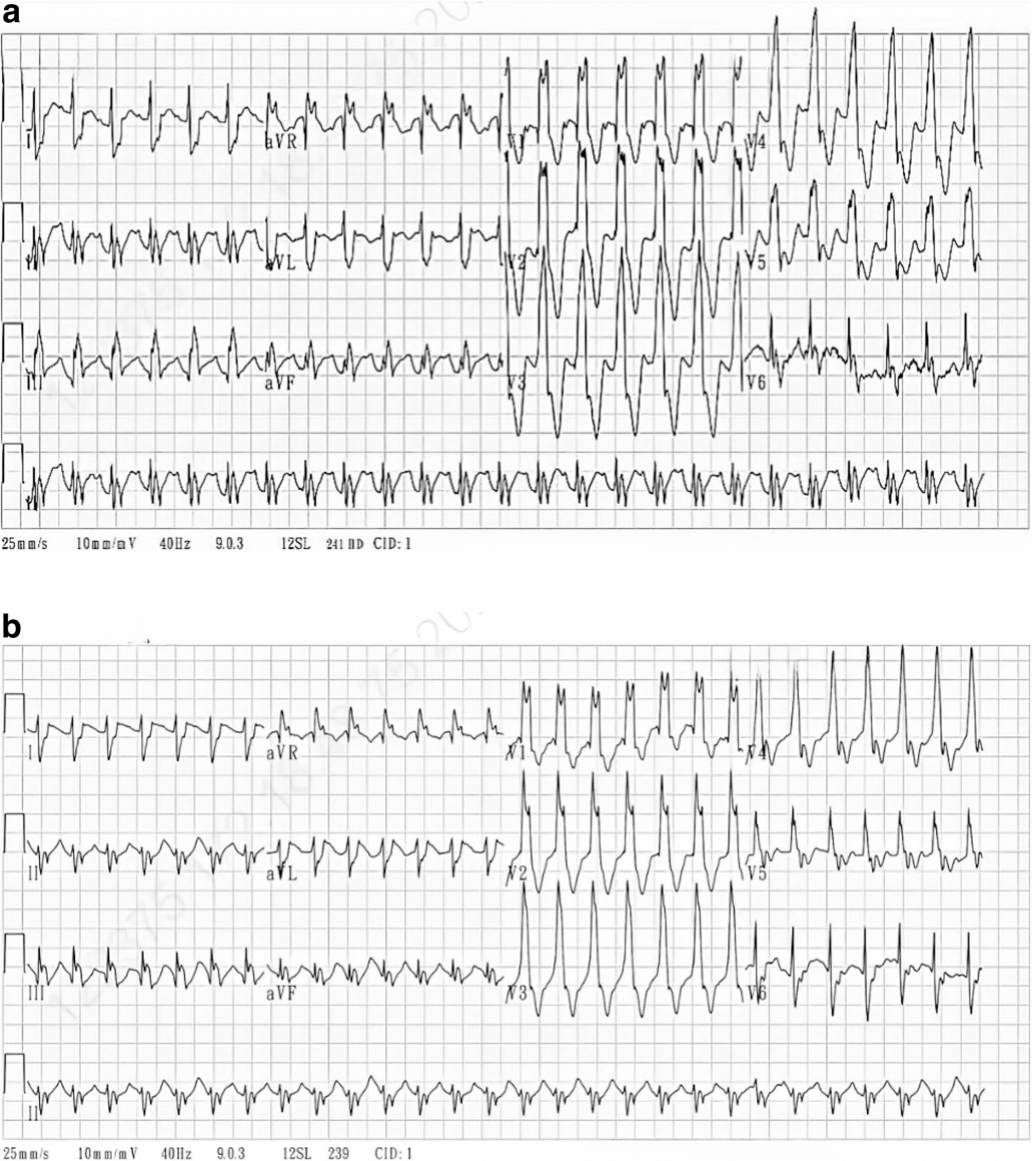
**a** An electrocardiogram taken upon admission. **b** An electrocardiogram taken after two doses of remdesivir were administered. Comparison of ECG before and after the use of remdesivir reveals the development of left posterior fascicular block, QTc prolongation, and QRS widening

Remdesivir therapy was initiated, with infusions administered on two consecutive days, each over 30 min, before the course was stopped. Two hours after the first infusion, hypotension with poor perfusion developed. His vital signs were as follows: heart rate 134/min, blood pressure, 75/42 mmHg; and CVP, 24 mmHg. The patient was intubated with ventilator support and catecholamine escalation. Blood culture yielded *Escherichia coli*, and the antibiotics were subsequently upgraded to Amikacin, Teicoplanin and Meropenem. Fluid resuscitation and component therapy were initiated for sepsis and disseminated intravascular coagulation. The second dose of remdesivir was administered the following day. The hypotension and lactatemia persisted and were refractory to inotropes. ECG revealed frequent premature ventricular contractions, the development of left posterior fascicular block, QTc prolongation from 445ms to 510ms, and QRS widening from 138ms to 182ms (Fig. 4a-b). A bedside ultrasound revealed enlarged RV compressing LV, and decreased LVEF (48%). Owing to profound shock and multiorgan failure, VA-ECMO was initiated.

Abdominal computed tomography revealed pneumoperitoneum, indicating IAI and bowel perforation. A PD catheter was inserted for drainage of ascites and peritoneal dialysis. Under strong antibiotic coverage and aggressive fluid resuscitation, his vital signs gradually stabilized, and he could be separated from ECMO 5 days later.

The patient remained comatose even when the sedation was withdrawn. In addition, the pneumonia and heart failure worsened. We continued medical treatment, including the escalation of catecholamines and antibiotics. Explorative laparotomy was postponed due to critical condition of the patient. The patient died 28 days after the ECMO removal.

**Fig. 5:**
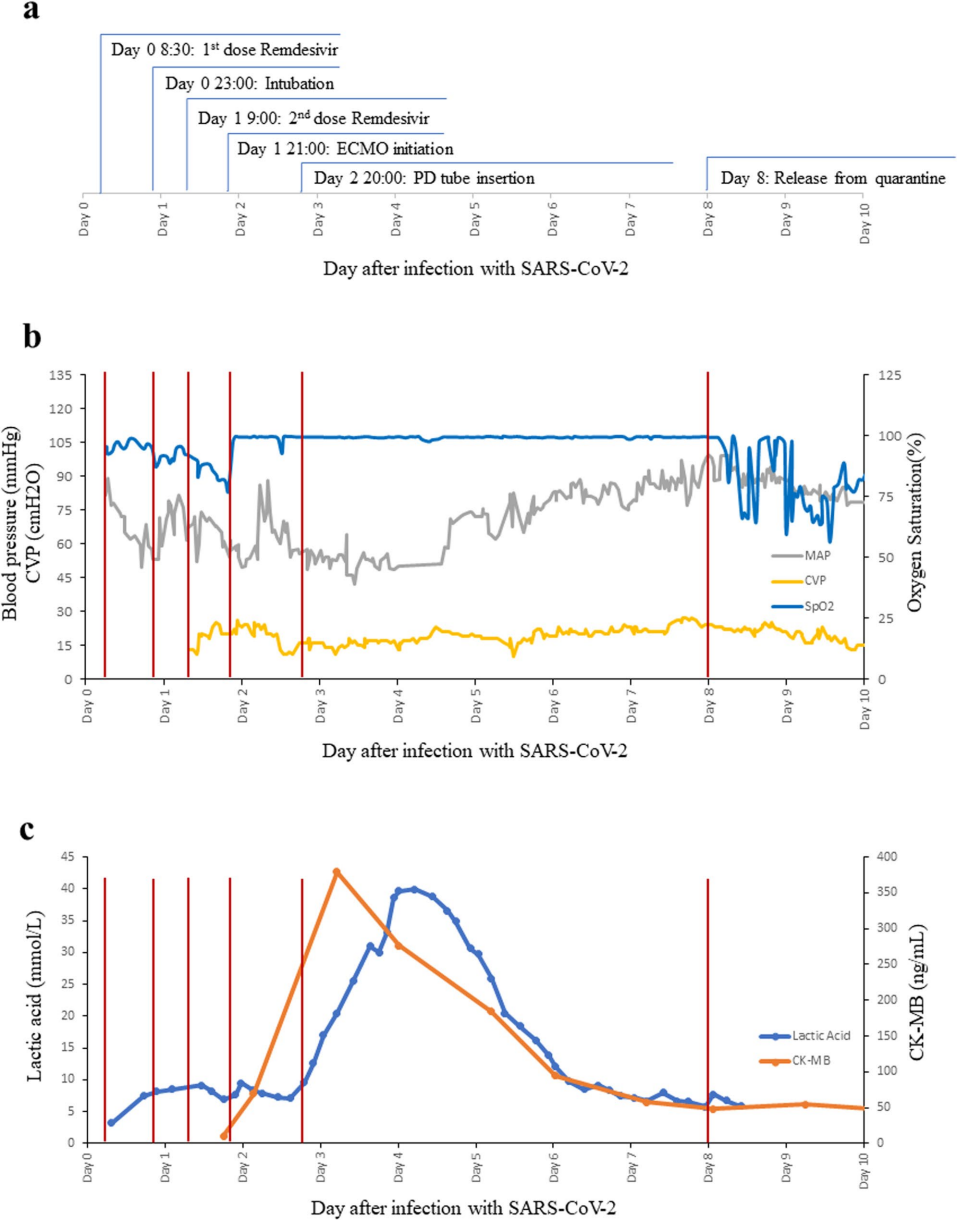
**a**-**c** Clinical details and changes in lab data over time in Patient 2 after infection with SARS-CoV-2 and the administration of remdesivir. Day 0 represents the day of confirmed SARS-CoV-2 infection. MAP, mean arterial pressure; CVP, central venous pressure; PD, peritoneal dialysis

The clinical details and changes in lab data over time in Patient 2 after infection with SARS-CoV-2 and the administration of remdesivir were summarized in Fig. 5a-c.

## Discussion and conclusions

Both patients had complex CHD and IAI simultaneously when they tested positive for SARS-CoV-2 with a high viral load. Prior to remdesivir infusion, oxygen demand was modest, and both maintained blood pressure and oxygen saturation. Within three hours of remdesivir infusion, however, cardiogenic shock developed, and manifested as high CVP, impaired heart contractility, and high lactate levels. The hemodynamic deterioration was refractory to supportive care and vasoactive therapy. Electrocardiographic abnormalities were also noted, including QRS widening and, in one patient, a left posterior fascicular block.

The clinical course and temporal correlation raise the possibility that remdesivir contributed to myocardial suppression. Nonetheless, causality cannot be established from these two cases. Both children had severe sepsis and systemic inflammation, which are themselves well-recognized causes of myocardial dysfunction. Sepsis-induced cardiomyopathy arises from a multifactorial process involving cytokine-mediated negative inotropy (e.g., TNF-α, IL-1β, IL-6) [26, 27], nitric oxide overproduction [27], mitochondrial dysfunction with impaired oxidative phosphorylation [26–28], and microvascular dysregulation [26, 27].

**Fig. 6:**
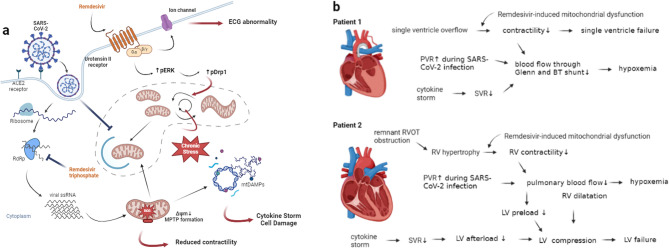
**a** Schema of the proposed model elucidating susceptibility to remdesivir-induced mitochondrial dysfunction due to pathological mitochondrial adaptation in patients with congenital heart disease. Adapted from “Coronavirus Replication Cycle”, by BioRender.com (2024). Retrieved from https://app.biorender.com/biorender-templates. **b** Brief summary of the pathophysiology of cardiogenic shock in these two cases based on the model presented in Fig. 6a, created with BioRender.com. RdRP, RNA-dependent RNA polymerase; MPTP, mitochondrial permeability transition pore; DAMP, damage associated molecular pattern; Δψm, inner membrane potential; pERK, phosphorylated ERK; pDrp1, phosphorylated Drp1; PVR, pulmonary vascular resistance; SVR, systemic vascular resistance; RVOT, right ventricular outflow tract; LV, left ventricle; RV, right ventricle.

As shown in Fig. 6a, SARS-CoV-2 infection may further exacerbate these processes. In the early phase of infection, SARS-CoV-2 can suppress the mitochondrial antiviral signaling pathway, thereby preventing host cells from producing type I and type III interferons and delaying the initiation of the antiviral immune response [29, 30]. This allows rapid viral proliferation. Once inside the mitochondrial matrix, viral RNA interferes with and hijacks mitochondrial biogenetic machinery [30, 31], leading to reduced mitochondrial redox potential and the assembly of the mitochondrial permeability transition pore (MPTP). Subsequent release of mitochondrial components into the cytoplasm acts as damage-associated molecular patterns (DAMPs), which bind to pattern-recognition receptors (PRRs) and amplify cytokine storm responses [32]. Normally, mitophagy selectively removes dysfunctional mitochondria as a critical quality-control mechanism. However, SARS-CoV-2 has been shown to prevent autophagosome formation and block mitophagy [33, 34], resulting in the accumulation of fragmented, dysfunctional mitochondria that further fuel inflammation. Several studies have also shown that SARS-CoV-2 can cause elevated pulmonary vascular resistance (PVR) [35–37].

As shown in Fig. 6b, certain CHD can lead to chronic ventricular overload. Under such stress, the high mitochondrial turnover rate ensures that the quality of mitochondria meets the needs of myocardial metabolism [38]. When combined with increased RV afterload, impaired mitochondrial quality control, and systemic inflammation, the compensatory capacity of the myocardium—particularly in single-ventricle circulation or chronically overloaded RVs—becomes severely compromised. In addition, low systemic vascular resistance (SVR), commonly observed in severe sepsis and cytokine storms, can further reduce diastolic pressure and impair coronary perfusion. This mismatch between myocardial oxygen supply and demand raises the risk of decompensated heart failure.

In this context, we postulate that remdesivir administration may have acted as the final tipping factor—the “last straw”—on top of multiple concurrent insults. Both patients were already vulnerable, with myocardium at the edge of decompensation through the previously described pathophysiological mechanisms.

Remdesivir has been reported to potentiate mitochondrial dysfunction and lead to cytotoxicity. Unlike anti-HIV nucleoside and nucleotide analogs, remdesivir does not directly inhibit mitochondrial DNA polymerase. It has weak inhibitory activity toward mitochondrial RNA polymerase and therefore does not significantly affect mitochondrial protein homeostasis [39]. Instead, remdesivir has been identified as a selective, partial agonist for the urotensin-II receptor (UTS2R). The half-maximal effective concentration (pEC50) of remdesivir was estimated to be 4.89 ± 0.03 (EC50 = 13 ± 0.9 µM), with the working range of agonistic effects starting at 1 µM based on the response curve [15]. UTS2R, as a GPCR, in turn activates heterotrimeric G proteins, which leads to dissociation of Gα and Gβγ subunit complexes [40]. Possibly through impaired regulation of gene expression or trafficking of ERG potassium channels, field potential, which correlates closely with the QT interval, is prolonged [15]. Remdesivir can also alter electrophysiological properties by reducing the spontaneous firing rate, which may disrupt conduction and lead to ventricular premature complexes, and by lowering the diastolic depolarization rate, which may contribute to QRS widening [14]. In addition, through binding to UTS2R, remdesivir can activate AKT/ERK axis [15]. Phosphorylation of ERK can in turn phosphorylate mitochondrial fission dynamic-like protein 1 (Drp1) [41]. Thus, Remdesivir induces mitochondrial fragmentation and dysfunction [14]. These effects are not tissue-specific [42] and are reversed by Mdivi-1, an inhibitor of Drp1 [14]. Augmented mitochondrial dysfunction may manifest as elevated lactic acid levels, cytokine storm, and impaired contractility, as observed in the two cases.

What makes this hypothesis more plausible is the pharmacokinetic profile of remdesivir, which aligns with the temporal correlation between drug administration and onset of shock. Remdesivir undergoes sequential hydrolysis to the nucleoside metabolite GS-441,524, which is transmitted intracellularly and converted into the active metabolite remdesivir triphosphate. Remdesivir has a plasma half-life of approximately one hour. Following a single dose of 200 mg of remdesivir administered, the area under the concentration-time curve from 0 to 24 h (AUC_0−24_) is 4.8 µM•h for remdesivir and 7.7 µM•h for GS-441,524 [43]. Remdesivir triphosphate has a longer half-life of up to 43 h [39, 44]. Mitochondrial dynamics occur on a timescale of minutes [45]. Clinically, peak remdesivir concentration is reached within two to three hours after infusion, which corresponds to the timing of cardiogenic shock in both patients. Additionally, based on the AUC_0−24_ and the working range of UTS2R agonistic effects of remdesivir, remdesivir-induced myocardial suppression could plausibly persist for at least one day or more. This temporal alignment is consistent with the sudden onset and protracted course of cardiogenic shock in both cases.

We acknowledge that no direct causal relationship can be established based on two cases. Our postulation is derived from clinical observation, temporal correlation, and existing experimental data. It remains entirely possible that the fatal outcomes were driven primarily by severe COVID-19, underlying CHD, and concurrent infection. More evidence from both clinical studies and mechanistic investigations will be required to validate this hypothesis.

Given that remdesivir has shown the greatest benefit in the early phase of infection, with relatively inconsistent effects on reducing mortality, its use in critically ill children with complex CHD and ongoing systemic inflammation warrants caution. In the present cases, the onset of symptoms and timing of remdesivir initiation suggest that the therapeutic window may already have passed, while the myocardium was highly vulnerable. Current dosing protocols are weight-based, and although prolonging infusion time may theoretically reduce peak-related toxicity, The overall effect is likely limited, as myocardial suppression induced by remdesivir administration may persist for more than one day. Reducing the weight-based dosage could compromise antiviral efficacy. Thus, in similar high-risk scenarios, withholding remdesivir may deserve consideration.

## Data Availability

No datasets were generated or analysed during the current study.

## Abbreviations

AUC: Area Under the Concentration–Time Curve
BT shunt: Blalock–Taussig shunt
CHD: Congenital Heart Disease
CPR: Cardiopulmonary Resuscitation
CRP: C–Reactive Protein
CT: Computed Tomography
CVP: Central Venous Pressure
CXR: Chest X–Ray/Chest Radiograph
DAMPs: Damage–associated Molecular Patterns
Drp1: Dynamin–related Protein 1
ECG: Electrocardiogram
ECMO: Extracorporeal Membrane Oxygenation
ERK/pERK: Extracellular Signal–regulated Kinase/phosphorylated ERK
FDA: U.S. Food and Drug Administration
IAI: Intra–abdominal Infection
IL: 6–Interleukin–6
LV: Left Ventricle
LVEF: Left Ventricular Ejection Fraction
MAP: Mean Arterial Pressure
MAPCAs: Major Aortopulmonary Collateral Arteries
MPTP: Mitochondrial Permeability Transition Pore
PD: Peritoneal Dialysis
PRRs: Pattern Recognition Receptors
PVR: Pulmonary Vascular Resistance
QTc: Corrected QT Interval
RdRP: RNA–dependent RNA Polymerase
RV: Right Ventricle
RVOT: Right Ventricular Outflow Tract
RT: PCR–Reverse Transcriptase–Polymerase Chain Reaction
SaO₂: Arterial Oxygen Saturation
SvO₂: Mixed Venous Oxygen Saturation
SVR: Systemic Vascular Resistance
TNF: α–Tumor Necrosis Factor alpha
UTS2R: Urotensin–II Receptor
VA: ECMO–Venoarterial Extracorporeal Membrane Oxygenation
Δψm: Mitochondrial Inner Membrane Potential
